# Phylogenetic Characterization and Seroprevalence of *Senecavirus* A from Swine Farms in Taiwan

**DOI:** 10.3390/ani15192786

**Published:** 2025-09-24

**Authors:** Cheng-Ju Pan, Kuo-Jung Tsai, Jen-Chieh Chang, Ming-Chung Deng, Nien-Nung Lin, Kelly M. Lager, Ian D. Robertson, Yu-Liang Huang

**Affiliations:** 1 Veterinary Research Institute, Ministry of Agriculture, New Taipei City 25158, Taiwan; cjpan@mail.nvri.gov.tw (C.-J.P.); 2 Animal and Plant Health Inspection Agency, Ministry of Agriculture, Taipei 100060, Taiwan; 3 National Animal Disease Center, Agricultural Research Service, United States Department of Agriculture, Ames, IA 50010, USA; 4 School of Veterinary Medicine, College of Environment and Life Sciences, Murdoch University, Perth, WA 6150, Australia

**Keywords:** *Senecavirus* A, seroprevalence, phylogenetic analysis, Taiwan, vesicular disease

## Abstract

*Senecavirus* A (SVA) is an emerging threat to swine populations due to its potential to cause vesicular lesions, which are difficult to differentiate from other vesicular diseases of swine such as foot and mouth disease (FMD), requiring significant resources for differential diagnosis. The first Taiwanese isolate of SVA was identified in 2006, although the first clinical case was not reported until 2012. The genetic characteristics and seroprevalence of SVA in Taiwan remain unclear. This study aimed to assess the seroprevalence and genetic diversity of SVA in nursery/weaned swine and finisher swine on Taiwanese pig farms. Phylogenetic analysis of seven Taiwanese SVA isolates revealed clustering into groups I and II. The 2006 and 2012 isolates shared 95.5% and 95.7% identity, respectively, with an early USA strain (MT360258), while more recent strains collected between 2018 and 2022 exhibited 95.7–98.8% identity with a 2020 USA strain (MZ733977). Serological analysis of swine from 300 farms showed significantly higher herd-level seroprevalence in nursery/weaned swine (53%) than finisher swine (6.7%). Furthermore, comparative analysis of nine known B cell epitopes showed high sequence conservation across Taiwanese and global strains. These findings provide important baseline data on the genetic diversity and seroprevalence of SVA in Taiwan and support the development of improved surveillance strategies for this emerging swine pathogen.

## Simple Summary

*Senecavirus* A is a virus that affects pigs and can cause blisters, often leading to confusion with more serious diseases such as foot and mouth disease. This study was designed to compare viral strains from Taiwan with other countries and how they are distributed in Taiwan. We found that the viral strains in Taiwan are genetically similar to those found in the United States of America. Furthermore, the seroprevalence data indicated that exposure to SVA is widespread in Taiwan. These findings give important information to help farmers, veterinarians, and officials monitor and control the disease more effectively.

## 1. Introduction

*Senecavirus* A (SVA), also known as Seneca Valley virus, is a non-enveloped, single-stranded positive-sense RNA virus belonging to the family *Picornaviridae* and the genus *Senecavirus* [1]. It is currently the only member within this genus to infect swine. A distinct member of the genus, Senecavirus cetus, was recently identified in cetaceans and represents a possible expansion of the host range within the genus, though it has not been reported in pigs [2]. The SVA genome is approximately 7.2 kb in length and encodes a polyprotein that is processed into four structural proteins (VP1, VP2, VP3, and VP4) and eight non-structural proteins. SVA was first identified in 2002 as a contaminant in cell culture media and was considered a harmless virus [1,3].

Since its initial discovery, retrospective analyses have revealed that SVA had been silently circulating in North American swine populations from as early as the late 1980s [4]. The virus was sporadically detected in various clinical samples between 1988 and 2005, though its pathogenic role was unclear at the time [4]. A 2007 case in Canada, where vesicular lesions were observed in pigs transported to the USA, raised suspicions about SVA as a potential vesicular disease agent [5]. Similarly, an isolated case in Indiana, USA, in 2012, also linked SVA to vesicular signs [6]. However, it was not until large-scale outbreaks occurred in Brazil (2014) and the USA (2015) that SVA was recognized as an emerging pathogen responsible for vesicular disease in swine [7,8]. The virus was soon identified in several other countries, including the People’s Republic of China (PROC), Colombia, and Thailand, demonstrating its potential for global spread [9,10,11]. These outbreaks were associated not only with vesicular lesions in pigs but also with epidemic transient neonatal losses (ETNL) [12].

In the USA, serological studies indicate that SVA has circulated in swine populations for over 30 years [13]. The clinical signs of SVA infection, including lethargy, anorexia, fever, lameness, and the presence of vesicles on the snout, oral cavity, and coronary band which are indistinguishable from those of other vesicular diseases, including FMD, swine vesicular disease (SVD), vesicular stomatitis (VS), and vesicular exanthema of swine (VES) [8,14].

Molecular epidemiological studies have classified SVA strains into distinct phylogenetic clades [15], with evidence indicating that contemporary isolates are generally more capable of inducing vesicular disease in pigs than historical isolates [16]. The widespread circulation of SVA and its ability to induce lesions indistinguishable from those caused by foot and mouth disease virus (FMDV) present significant challenges to veterinary diagnostics and disease control [5]. In non-FMD-endemic regions, such as Taiwan, where strict surveillance measures are in place to maintain FMD-free status, the presence of SVA as a vesicular disease agent highlights the importance of continued monitoring and investigation into its epidemiology and genetic characteristics [17].

In Taiwan SVA was first isolated from blood-contaminated effluent collected from a truck used to transport pig carcasses to a rendering plant in 2006. However, the first clinical detection of SVA was not reported until 2012, when a contracted veterinarian at a farrow-to-finish swine farm in Hualien County reported suspected vesicular lesions on the coronary bands of finisher swine to the Local Animal Disease Inspection Authority (LADIA). Samples were collected by veterinarians from LADIA and submitted to the Veterinary Research Institute (VRI) under the Ministry of Agriculture in Taiwan for testing. All samples tested negative for FMD, SVD, VS, and VES, but SVA was identified. Subsequently, SVA was isolated from two clinical cases of pigs with vesicular lesions in Taiwan. In 2018, a veterinary meat inspector at an abattoir in Tainan City also observed vesicular lesions in swine in lairage and reported this finding to the Animal and Plant Health Inspection Agency. In 2020, the owner of a farrow-to-finish swine farm in Tainan City also reported vesicular lesions in finisher swine to the LADIA. Samples from both cases were collected by veterinarians of the LADIA and sent to the VRI for testing for FMD, SVD, VS, VES, and SVA. SVA was isolated from these samples, while agents of other vesicular diseases were not detected and consequently these diseases were ruled out from the differential diagnoses. Although SVA has not been associated with widespread or devastating economic losses comparable to those caused by FMD, its emergence as a vesicular disease pathogen that results in clinical signs almost identical to FMD highlights the need for enhanced surveillance and understanding of its epidemiology [14,18].

Among vesicular diseases of swine, foot and mouth disease (FMD) is prominent due to its highly contagious nature and devastating economic consequences, posing significant threats to the swine industry [19]. After more than 23 years of concerted effort, Taiwan achieved FMD-free status without vaccination for the Taiwan, Penghu, and Matsu regions in 2020 [20]. To maintain this status, all vesicular disease cases in swine must be treated as suspected FMD until confirmed otherwise through laboratory testing. This underscores the critical importance of understanding the epidemiology of the emerging vesicular disease pathogen, SVA, in Taiwan. However, there is limited information on the prevalence and genetic characteristics of SVA in the Taiwanese swine population. Establishing baseline epidemiological and phylogenetic data for SVA is essential for improving understanding of its infection dynamics and assessing its potential impact on the swine industry [21].

To address these gaps, this study was designed to: investigate the phylogenetic and epidemiological characteristics of SVA in Taiwan by determining the animal-level and farm-level seroprevalence of SVA in swine across the country; and characterize the genetic profile of Taiwanese SVA isolates. By comparing these isolates with those from other countries, this study seeks to provide critical insight into the epidemiology of SVA in Taiwan and support the development of effective surveillance and control strategies.

## 2. Materials and Methods

### 2.1. Sample Collection

A total of 7 SVA-positive samples were included in this study for sequencing and phylogenetic analysis between 2006 and 2022. Samples archived from previous investigations of cases of vesicular disease were also included in this study for sequencing and phylogenetic analysis. These samples had all been confirmed, at the time of collection, to be negative for FMD, SVD, VS and VES. These archived samples included vesicular epithelial samples collected from cases of vesicular lesions in finisher pigs aged 20–26 weeks in Taiwan in 2012, 2018, and 2020. Additionally, a 2006 strain (ID: HC061119) was isolated from blood-contaminated effluent collected from a truck used for transporting pig carcasses to a rendering plant. Two SVA isolates from 2021 and 2022 were also obtained from oral swab samples collected from swine that had initially tested positive for FMDV NSP antibodies in a separate FMD surveillance program. These FMD test results were subsequently confirmed as false positives based on negative results in virus neutralization tests (VNT) and nucleic acid detection for FMDV. Specifically, one strain from 2021 (ID: 1102577) was isolated from oral swabs collected from 20–26-week-old pigs in a farm in Tainan City, and one strain from 2022 (ID: 1111570) was isolated from oral swabs collected from a 20-week-old pig in a farm in Pingtung County. Additionally, another strain from 2022 (ID: 1111388) was isolated from lymph node tissue collected from a dead 8-week-old pig from a farm in Pingtung County. According to records from a veterinarian of the LADIA, this pig had exhibited agonal respiration (gasping) prior to death. Testing conducted at the VRI identified PRRSV and SVA from the collected tissues, while FMDV and other major swine diseases were ruled out. Nucleic acid extraction was conducted within one day of sample collection for all oral swab and tissue samples. For the blood-contaminated effluent sample, nucleic acid extraction was performed immediately after virus isolation. All cDNA products were stored at −80 °C until subsequent sequencing and analysis in this study.

### 2.2. RNA Extraction

Oral swab samples were placed in 3 mL of Minimum Essential Media (MEM; Thermo Fisher Scientific, Waltham, MA, USA) immediately after collection. At the laboratory, the cotton swab was pressed against the tube wall to extract the liquid and the samples centrifuged at 3000 rpm and the supernatant collected. To prepare a 10% (*w*/*v*) homogenate, vesicular epithelial samples were homogenized and mixed with 3 mL of MEM supplemented with 5% fetal calf serum and 1% antibiotics. The homogenates were centrifuged at 3000 rpm, and the supernatant collected. The supernatants derived from swabs and tissue homogenates were subjected to automated nucleic acid extraction using the TANBead Nucleic Acid Extraction Kit (Taiwan Advanced Nanotech Inc., Taoyuan City, Taiwan) according to the manufacturer’s instructions. The extracts were eluted in 100 μL of elution buffer and subsequently used for both real-time reverse transcription PCR (RRT-PCR) detection and reverse transcription PCR (RT-PCR) amplification of the complete SVA genome.

### 2.3. RRT-PCR

A real-time reverse transcription PCR (RRT-PCR) targeting the conserved region of the VP1 gene was performed according to the method described by Dall Agnol et al. (2017) [22]. Reactions were performed using the LightCycler^®^ 480 system (Roche Diagnostics GmbH, Mannheim, Germany) in a 20 µL reaction mixture containing 4 µL of LightCycler^®^ 480 Probes Master (2×; Roche Diagnostics, Mannheim, Germany), 0.1 µL of AMV Reverse Transcriptase (10 U/µL; Promega Corporation, Madison, WI, USA), 1 µL each of forward and reverse primers (20 µM), 0.5 µL of probe (10 µM), 3 µL of RNA template, and 10.4 µL of DEPC-treated water (Thermo Fisher Scientific, Waltham, MA, USA). The thermal program was as follows: 50 °C for 30 min (1 cycle), 95 °C for 3 min (1 cycle), followed by 45 cycles of 95 °C for 15 s and 60 °C for 1 min, and a final step at 40 °C for 30 s.

### 2.4. Amplification of the SVA Genome and Sequencing

To amplify the SVA genome, we designed seven primer pairs based on the sequence of strain USA/KS15-031348/2015 (MN233025), as detailed in Table 1. RT-PCR was performed with the Phusion Green Hot Start II High-Fidelity DNA Polymerase kit (Thermo Fisher Scientific) in a GeneAmp PCR System 2700 thermal cycler (Applied Biosystems, Foster City, CA, USA). The thermal cycling conditions were as follows: 42 °C for 40 min, 95 °C for 5 min, followed by 35 cycles of 95 °C for 15 s, 55 °C for 15 s, and 72 °C for 30 s, with a final step at 40 °C for 30 s before holding at 4 °C.

The PCR products were purified from agarose gels and submitted for commercial sequencing service (Genomics, Taipei City, Taiwan). Sanger sequencing was performed using the BigDye Terminator v3.1 Cycle Sequencing Kit (Applied Biosystems, Foster City, CA, USA) with the same primers as in the PCR. The sequencing reaction was prepared according to the manufacturer’s instructions. The sequencing products were analyzed using an ABI 3730xl DNA analyzer (Applied Biosystems, Foster City, CA, USA). Each nucleotide position was confirmed by sequencing at least two times.

### 2.5. Sequence Identity and Phylogenetic Analysis

The full-length sequences and 4 structural proteins (VP1–4) of the 7 isolated SVA strains from this study were aligned with 18 reference sequences available in GenBank (https://www.ncbi.nlm.nih.gov/genbank/, accessed on 11 September 2024), as well as 12 additional sequences provided by the Agricultural Research Service (ARS), United States Department of Agriculture (USDA). These 18 GenBank sequences were selected to represent global diversity, including strains from the USA (2012–2020, *n* = 4), Canada (2011–2015, *n* = 3), Brazil (2015, *n* = 4), Colombia (2016, *n* = 1), China (2016–2018, *n* = 4), and Thailand (2016, *n* = 2), based on their geographic origin and collection year. In addition to full-genome phylogenetic analysis, we also analyzed all four structural protein genes (VP1–VP4), which encode the viral capsid and are commonly associated with host immune responses [23]. The inclusion of all four VP genes provided a more comprehensive understanding of capsid-related variation.

A phylogenetic tree was constructed using the Maximum Likelihood method in MEGA XI software (Pennsylvania State University, PA, USA) [24], applying the Tamura–Nei substitution model, which was identified as the best-fitting model using the function for substitution model selection based on the lowest Bayesian Information Criterion score. The analysis was performed with 1000 bootstrap replicates to determine the genetic relationships between the Taiwanese SVA strains and those from other countries.

### 2.6. Epitope Sequencec Analysis

In addition, amino acid sequence comparisons of known B cell epitopes were conducted by MEGA XI software. A total of nine previously identified linear B cell epitopes within VP1, VP2, and VP3 regions previously identified through monoclonal antibody-based methods were analyzed [25,26,27]. The selected epitopes included ^21^GELAAP^26^ within VP1; ^12^DRVITQT^18^, ^71^WTKAVK^76^, ^98^GGAFTA^103^, ^150^KSLQELN^156^, ^177^SLGTYYR^183^, ^248^YKEGAT^253^ and ^266^SPYFNGL^272^ within VP2; ^192^GWFSLHKLTK^201^ within VP3. Aligned amino acid sequences from seven Taiwanese isolates and 30 representative global SVA strains were analyzed to identify potential amino acid substitutions within these epitope regions.

### 2.7. Serological Surveillance

Serum samples were collected between January and December 2021 across 19 administrative divisions (counties/cities) in Taiwan, covering five major regions: northern, central, southern, eastern, and the offshore islands, without targeting any specific season. Three administrative divisions—Keelung City, Taipei City, and Lienchiang County—were excluded from this sampling due to the absence of representative swine populations. The sample size was determined using the online Epitools sample size calculator (https://epitools.ausvet.com.au/, accessed on 26 October 2024), assuming an expected farm-level seroprevalence of 7.4% as reported in a 2022 study conducted on SVA seroprevalence in swine older than 20 weeks of age in the USA [28], with a 5% precision and a 95% confidence interval. Based on a 2020 survey by the Ministry of Agriculture, Taiwan, there were a total of 6497 swine farms in Taiwan. Using this number as the population of farms at risk, at least 106 swine farms were required to be sampled; however, a total of 300 farms were subsequently sampled to increase representativeness and reliability of the study. Serum samples were collected from at least 14 swine on each selected farm, a number calculated to be 95% confident of detecting at least three seropositive animals assuming a within-herd animal-level seroprevalence of 20%. If there were fewer than 14 swine on the selected farm, all swine on that farm were sampled. The selected swine were monitored longitudinally, with two serum samples collected. The first sample set was collected during the nursery and weaner (6–12 weeks of age, post-weaning) stage, and the second set was collected from the same pigs during the finisher stage (20–26 weeks). This sampling design allowed for the detection of changes in neutralizing antibody status between the two stages (age groups). Serum samples were heat-inactivated at 56 °C for 30 min, stored at −20 °C and tested within one week of collection.

### 2.8. Anti-SVA Neutralizing Anitibody Assay

Antibodies to SVA were detected using the VNT assay. Serial two-fold dilutions of serum were carried out and mixed with 100 TCID_50_ of the W107-0691 Taiwanese strain. After incubation at 37 °C for 1 h, the BHK-21 cells were then added and the mixture incubated at 37 °C for 48 h. Neutralizing titers were defined as the highest serum dilution that completely inhibited the cytopathic effect. Samples with neutralization antibody titters ≥1:64 were classified as positive [29]. Farms were classified as seropositive for SVA if the farm had more than two anti-SVA VNT-positive pigs of the same stage (age-group).

### 2.9. Statistical Analysis

To compare the farm-level seroprevalence of SVA among different groups, pairwise comparisons were conducted using GraphPad Prism 10.4.1 (GraphPad Software, LLC, San Diego, CA, USA). Odds ratios (ORs) and their 95% confidence intervals (CIs) were also calculated to compare the seroprevalence between the regions. The OR calculations followed the methodology described in Statistical Methods in Epidemiology, Kahn et al. (1989) [30]. The SVA seroprevalence in administrative divisions was displayed using maps created with QGIS 3.28.7 (https://qgis.org/ accessed on 26 October 2024, an Open Source Geospatial Foundation Project headquartered in Beaverton, OR, USA).

## 3. Results

### 3.1. Phylogenetic Analysis of Full-Length Sequence in Taiwanese SVA Strains

#### 3.1.1. Full-Length Sequence Analysis

Seven Taiwanese strains of SVA obtained from previous sampling investigations and the FMD surveillance program were sequenced and analyzed in this study. Based on full-length sequences analysis, nucleotide and amino acid sequences the identities of the 7 Taiwanese isolates ranged from 92.2% to 99.6% and 96.0% to 99.0%. The sequences showed the highest nucleotide and amino acids sequence identities to the recent USA strain SVA-USA-OH-NADC4-2020 (MZ733977) with identities ranging from 92.4% to 98.8% and 95.7% to 98.4%, respectively.

**Figure 1:**
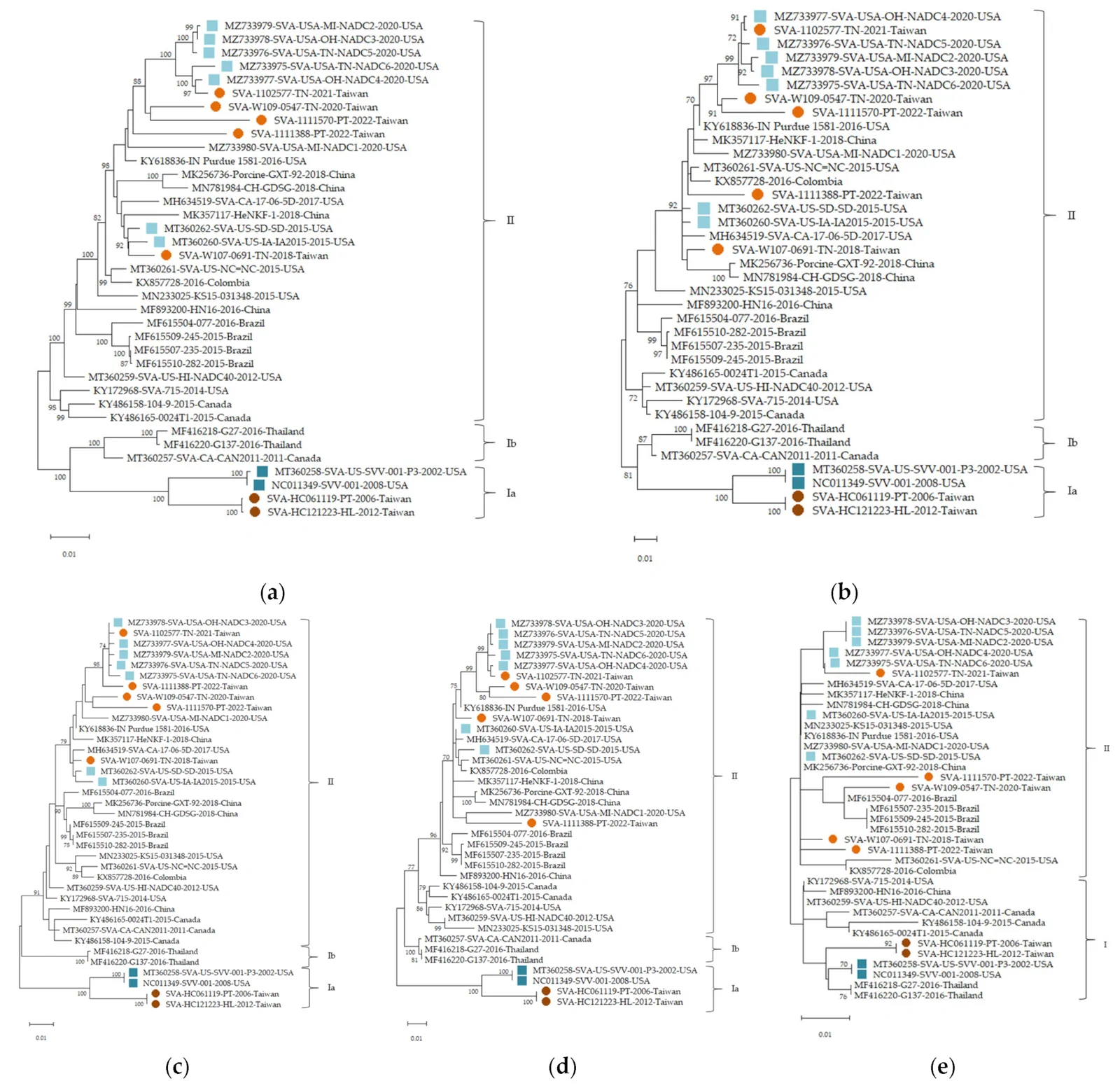
Maximum likelihood phylogenetic trees of *Senecavirus* A strains constructed using MEGA XI with 1000 bootstrap replicates based on (**a**) full-length sequence, (**b**) VP1, (**c**) VP2, (**d**) VP3, and (**e**) VP4 sequences. Bootstrap values above 70% are shown at the nodes. Solid circles indicate SVA strains from Taiwan identified in this study, and solid squares represent strains closely related to the Taiwanese viruses based on full-length sequence phylogenetic analysis. Strains in dark colors belong to group Ia, and those in light colors belong to group II. Among the Taiwanese strains, those detected in 2006 and 2012 are colored brown, and those detected during 2018–2022 are colored orange. Closely related strains are colored dark blue if related to earlier Taiwanese strains and light blue if related to recent Taiwanese strains. The corresponding GenBank accession numbers for each Taiwanese strain are: HC061119 (PV002715), HC121223 (PV002716), W107-0691 (PV002717), W109-0547 (PV002718), 1102577 (PV002719), 1111388 (PV002713), and 1111570 (PV002714).

The full-length sequence phylogenetic tree revealed two groups among the Taiwanese isolates. The early isolates HC061119 (2006) and HC121223 (2012), which clustered phylogenetically with the prototype strain SVV-001, were classified into group Ia. The other five contemporary Taiwanese isolates collected after 2018, were classified into group II and clustered with USA strains from 2020 (Figure 1a). The mean nucleotide divergence (p-distance) within each group was 2.90% for group Ia, 1.57% for group Ib, and 3.27% for group II. The divergence between group Ia and Ib was 7.51%, and between group Ib and II was 6.60%, supporting their classification into distinct phylogenetic groups. HC061119-PT-2006 and HC121223 exhibited 95.1–95.7% nucleotide and 96.7–97.3% amino acid sequence identity with other members of group Ia. The five contemporary Taiwanese isolates obtained after 2018 showed the highest nucleotide and amino acid sequence identities to the recent USA strain SVA-USA-OH-NADC4-2020 (MZ733977), with identities ranging from 95.7% to 98.8% and 97.2% to 98.4%, respectively.

#### 3.1.2. Structural Protein Sequence Analysis

Maximum likelihood phylogenetic trees were constructed using full-length sequences of the VP1 (792 bp), VP2 (852 bp), VP3 (717 bp), and VP4 (213 bp) genes (Figure 1b–e). Across the VP1, VP2, and VP3 trees, three phylogenetic groups were consistently observed, and the bootstrap values of most nodes exceeded 70%. These groups showed topological consistency with the grouping patterns observed in the full-length sequence phylogenetic analysis result. Historical Taiwanese strains (HC061119 and HC121223) clustered within group Ia, alongside the prototype strain SVV-001. In contrast, Taiwanese isolates collected after 2018 (W107-0691, W109-05470, 1102577, 1111570, and 1111388) together with USA contemporary strains clustered within group II, indicating a shift toward contemporary virus lineages. The bootstrap support values of VP4 were generally lower, and clear separation into three groups was not observed.

#### 3.1.3. Epitope Sequence Analysis

As this study was primarily undertaken to provide a foundation for SVA genetic diversity and seroprevalence in Taiwan, no novel epitope prediction or experimental mapping were conducted. Amino acid sequence alignment of the nine linear B cell epitopes across seven Taiwanese and 30 representative global SVA strains revealed a high degree of conservation. Seven out of nine epitopes were completely conserved across all analyzed strains, including ^21^GELAAP^26^ in VP1; ^98^GGAFTA^103^, ^150^KSLQELN^156^, ^177^SLGTYYR^183^, ^248^YKEGAT^253^, and ^266^SPYFNGL^272^ in VP2; and ^192^GWFSLHKLTK^201^ in VP3. Two minor amino acid variations were observed in the VP2 ^12^DRVITQT^18^ epitope, three strains—MT360258, HC061119, and KY486158—contained a threonine (T) instead of isoleucine (I) at position 15, resulting in the sequence DRVTTQT. All other strains, including Taiwanese isolates, carried the DRVITQT variant. In the VP2 ^71^WTKAVK^76^ epitope, one Taiwanese strain (1111570) exhibited a unique substitution of glycine (G) for tryptophan (W) at position 71, resulting in WGKAVK. No other substitutions were detected in the remaining epitope regions among the analyzed strains.

### 3.2. Seroprevalence of SVA in Nursery/Weaner Stage and Finisher Stage

#### 3.2.1. Herd and Animal-Level Seroprevalence in Nursery/Weaned Swine

**Figure 2:**
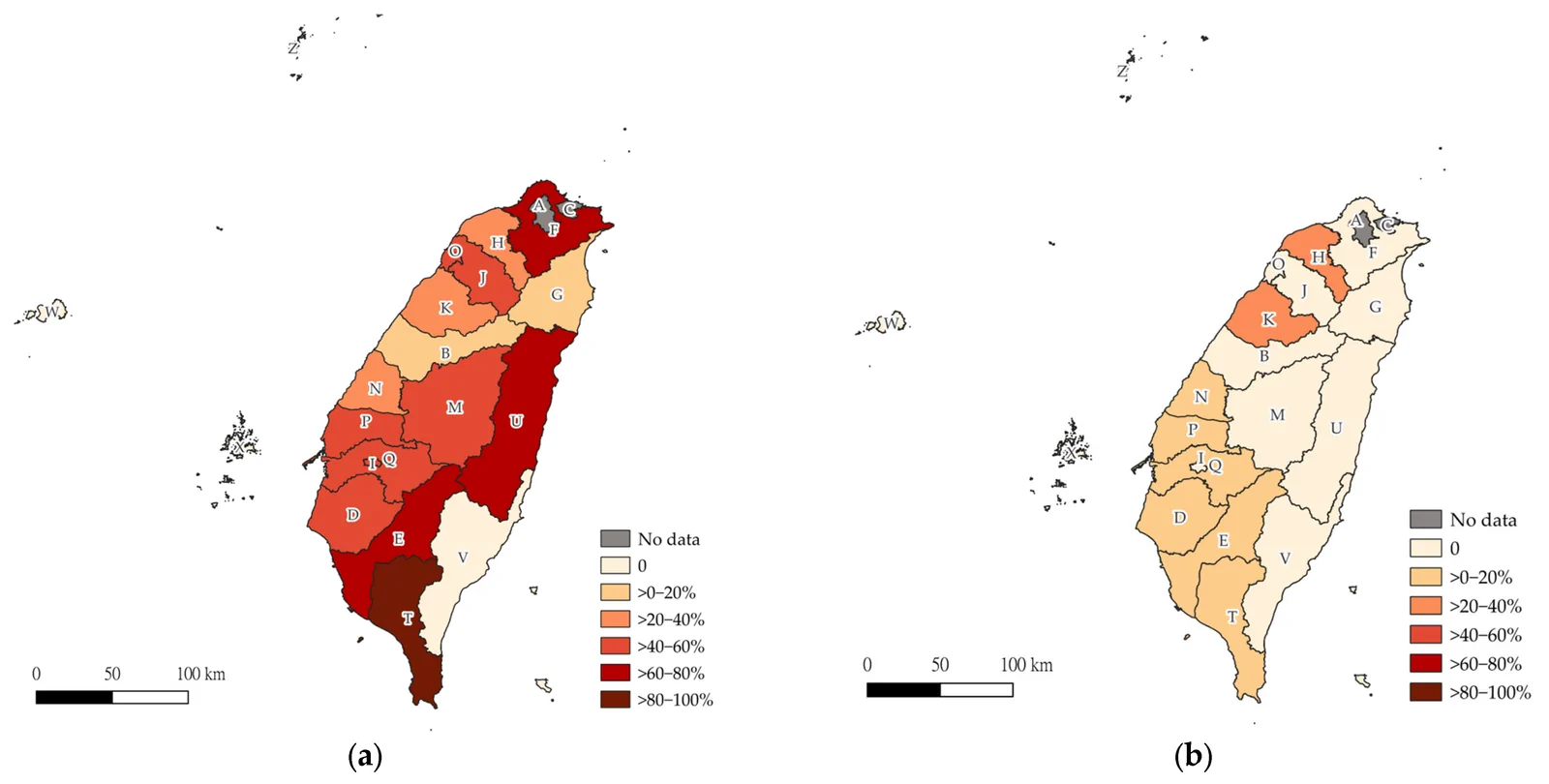
Geographical distribution of SVA farm-level seroprevalence across counties and cities in Taiwan during the nursery/weaner stage (**a**) and the finisher stage (**b**). The map shows the seroprevalence of *Senecavirus* A (SVA) across different counties in Taiwan. Each county is colored according to its county/city level seroprevalence (%), highlighting areas with high and low infection rates. Counties and cities in Taiwan are labeled with letter codes (A–Z), and only selected letters are assigned. The corresponding county/city names are as follows: A: Taipei City, B: Taichung City, C: Keelung City, D: Tainan City, E: Kaohsiung City, F: New Taipei City, G: Yilan County, H: Taoyuan City, I: Chiayi City, J: Hsinchu County, K: Miaoli County, M: Nantou County, N: Changhua County, O: Hsinchu City, P: Yunlin County, Q: Chiayi County, T: Pingtung County, U: Hualien County, V: Taitung County, W: Kinmen County, X: Penghu County, Z: Lienchiang County.

A total of 300 herds with pigs in the nursery/weaner stage were sampled and assayed. The results revealed that 159 farms were classified as seropositive with a farm-level seroprevalence of 53.0% (95% CI, 47.2–58.8). Overall, the farm-level seroprevalence in the nursery/weaner stage differed significantly across the five regions (*p* = 0.0001, χ^2^ = 22.88; df = 4,1). The farm seroprevalence of pigs in the nursery/weaner stage was highest in the southern region (65.7%, 95% CI, 57.0–73.7), followed by the central region (47.2%, 95% CI, 38.1–56.4). The eastern region had a similar farm-level seroprevalence of 37.5% (95% CI, 8.5–75.5) in the nursery/weaner stage to the northern region (37.0%, 95% CI, 19.4–57.6). No nursery/weaned swine from farms located on offshore islands were seropositive (95% CI, 0.0–45.9) (Table 2). The farm-level seroprevalence of the nursery/weaner stage in the southern region was significantly higher than that of the northern region (OR 3.3; 95% CI, 1.4–7.7, *p* < 0.01). All other regional farm-level seroprevalences for the nursery/weaner stage were not significantly different (Table 2 and Figure 2a).

Overall, there was a significant difference in the farm seroprevalence of nursery/weaned swine between the 19 counties/cities (*p* < 0.0001, χ^2^ = 53.01; df = 18,1). Notably, Pingtung County had the highest farm-level seroprevalence in the nursery/weaner stage (82.1%, 95% CI: 69.6–91.1), which was significantly higher than that in several other counties/cities, including Penghu County (*p* = 0.0399), Kinmen County (*p* = 0.0001), Taitung County (*p* = 0.0021), Changhua County (*p* = 0.0031),Tainan City (*p* = 0.0003), Changhua County (*p* < 0.0001), Yunlin County (*p* = 0.0017), Miaoli County (*p* = 0.0053), Taichung City (*p* = 0.0007), Taoyuan City (*p* = 0.0200), and Yilan County (*p* = 0.0002).

Of the 4508 nursery/weaned swine sampled, 1634 were seropositive, with an animal-level seroprevalence of 36.2% (95% CI, 34.8–37.7). The animal-level seroprevalence for these pigs also varied between regions (*p* < 0.0001, χ^2^ = 222.78; df = 4,1): southern region 46.3% (95% CI, 44.1–48.6), central region 31.4% (95% CI, 29.3–33.6), eastern region 27.5% (95% CI, 19.7–36.4), northern region 24.0% (95% CI, 19.9–28.4), and offshore islands 0.0% (95% CI, 0.0–2.4) (Table 2). The animal-level seroprevalence of pigs in the nursery/weaner stage in the central region was significantly higher than in the northern region (OR: 1.5, 95% CI, 1.1–1.9, *p* = 0.0033), and the southern region also showed significantly higher odds than the northern region (OR: 2.7, 95% CI, 2.2–3.5, *p* < 0.0001). Although no positive samples were detected in swine from offshore islands, the comparison with the northern region still yielded a statistically significant result on a Chi-square test (*p* < 0.0001); however, OR could not be calculated for this group (Table 2).

A county level comparison of the animal-level seroprevalence for nursery/weaned swine also showed an overall significant difference among the 19 counties/cities sampled (*p* < 0.0001, χ^2^ = 90.56; df = 18,1). Pingtung County exhibited the highest animal-level seroprevalence in this stage (57.5%, 95% CI: 54.1–60.9), which was significantly higher than that observed in several other counties/cities, including Penghu County (*p* < 0.0001), Kinmen County (*p* < 0.0001), Taitung County (*p* < 0.0001), Chiayi City (*p* < 0.0001), Chiayi County (*p* < 0.0001), Kaohsiung City (*p* = 0.0082), Tainan City (*p* < 0.0001), Changhua County (*p* < 0.0001), Yunlin County (*p* < 0.0001), Miaoli County (*p* < 0.0001), Nantou County (*p* < 0.0001), Taichung City (*p* < 0.0001), Hsinchu County (*p* < 0.0001), Hsinchu City (*p* < 0.0001), Taoyuan City (*p* < 0.0001), and Yilan County (*p* < 0.0001).

#### 3.2.2. Herd and Animal-Level Seroprevalence in Finisher Swine

In the 300 herds of finisher stage swine sampled and assayed, only 20 herds were classified as seropositive (farm-level seroprevalence of 6.7%; 95% CI, 4.1–10.1). The highest farm-level seroprevalence for finisher swine was observed in the central region (8.9%; 95% CI, 4.5–15.4), followed by the northern region (7.4%, 95% CI, 0.9–24.3) and southern region (5.2%; 95% CI, 2.1–10.5). No finisher herds were classified as seropositive in the eastern region or offshore islands (Table 3 and Figure 2b).

The farm-level seroprevalence for the finisher stage was similar between the five regions (*p* = 0.62, χ^2^ = 2.64; df = 4,1). Similarly, no significant differences were observed in the farm-level seroprevalences among the 19 counties/cities (*p* = 0.13, χ^2^ = 24.68; df = 18,1). Nevertheless, Miaoli County exhibited the highest finisher farm-level seroprevalence (37.5%, 95% CI: 8.5–75.5), which was significantly higher than that in several other counties/cities, including Kaohsiung City (*p* = 0.0359), Pingtung County (*p* = 0.0222), Tainan City (*p* = 0.0457), and Yunlin County (*p* = 0.0209).

A total of 4145 finisher swine were tested, and 192 pigs were classified as seropositive (animal-level seroprevalence of 4.6%; 95% CI, 4.0–5.3). The animal-level seroprevalence for finisher swine varied between the five regions (*p* = 0.0004, χ^2^ = 20.65; df = 4,1) with the highest animal-level seroprevalence in the central region (6.1%; 95% CI, 5.0–7.4) compared to the southern region (4.2%; 95% CI, 3.3–5.2), northern region (2.6%; 95% CI, 1.3–4.8), eastern region (0.9%; 95% CI, 0.0–4.7), and offshore islands (0.8%; 95% CI, 0.0–4.2) (Table 3). The animal-level seroprevalence of finisher swine in the central region was significantly higher than that of the offshore islands (OR 8.47; 95% CI, 1.17–61.23, *p* = 0.0055). All other regional animal-level seroprevalences in finisher swine were not significantly different (Table 3).

Overall, the animal-level seroprevalence of finisher swine was significantly different (*p* < 0.0001, χ^2^ = 486.63; df = 18,1) between the 19 counties/cities. Miaoli County had the highest animal-level seroprevalence (17.65%, 95% CI, 11.27–25.70), which was significantly higher than that observed in several other counties/cities, including Penghu County (*p* = 0.0080), Kinmen County (*p* < 0.0001), Hualien County (*p* = 0.0003), Taitung County (*p* = 0.0026), Chiayi City (*p* = 0.0142), Chiayi County (*p* = 0.0007), Kaohsiung City (*p* < 0.0001), Pingtung County (*p* < 0.0001), Tainan City (*p* < 0.0001), Changhua County (*p* < 0.0001), Yunlin County (*p* < 0.0001), Nantou County (*p* = 0.0013), Taichung City (*p* < 0.0001), Hsinchu County (*p* < 0.0001), Hsinchu City (*p* = 0.0080), New Taipei City (*p* = 0.0011), and Yilan County (*p* < 0.0001).

#### 3.2.3. Comparison of Seroprevalence Between Nursery/Weaner Stage and Finisher Stage

The farm-level seroprevalence of nursery/weaned swine (53.0%; 95% CI, 47.2–58.8) was significantly higher than that of finisher swine (6.7%; 95% CI, 4.1–10.1, *p* < 0.0001). Similarly, the animal-level seroprevalence of nursery/weaned swine was significantly higher than that of finisher swine (36.2%; 95% CI, 34.8–37.7% for nursery/weaned swine vs. 4.6%; 95% CI, 4.0–5.3% for finisher swine, *p* < 0.0001).

To further explore the relationship between seroprevalence in the nursery/weaner stage and finisher stage, the results were classified into four patterns: (1) Pattern I, which were herds that contained swine that were seropositive in both the nursery/weaner stage and finisher stage (*n* = 14); (2) Pattern II, which were herds that contained swine that were seropositive in the nursery/weaner stage but seronegative in the finisher stage (*n* = 145); (3) Pattern III, which were herds that contained swine that were seronegative in the nursery/weaner stage but seropositive in the finisher stage (*n* = 6); and (4) Pattern IV, which were herds that contained swine that were seronegative in both stages (*n* = 135). There was no significant difference in the overall farm-level seroprevalence between nursery/weaned swine and finisher swine among all patterns (*p* = 0.11, χ^2^ = 2.49; df = 1; OR 2.17, 95% CI, 0.81–5.82). Notably, RRT-PCR testing for SVA was conducted among the six herds classified as Pattern 3 (those that had swine that seroconverted), and three of these herds tested positive for SVA RNA.

## 4. Discussion

The phylogenetic analysis of SVA isolates from Taiwan revealed distinct clustering patterns corresponding to group Ia and II, consistent with a previous study [15]. HC061119 and HC121223, belonging to group Ia, showed high nucleotide identity (95.5% and 95.7%) with US-SVV-001-P3-2002, a strain with close phylogenetic relationship to the earliest characterized SVA viruses in the USA [15]. This suggested that the Taiwanese historical strains may have originated from an early lineage of SVA. Furthermore, the five more recent Taiwanese strains (2018–2022) exhibited higher nucleotide (95.7–98.8%) and amino acid (97.2–98.4%) sequence identity with SVA-USA-TN-NADC5-2020 (MZ733977), indicating the potential influence of international animal transport on viral evolution. Similar clustering of recent isolates within group II has been observed in other countries, emphasizing the global spread and diversification of SVA. Another study further supported the evolutionary trends of SVA strains, reporting genetic differences between the SVV 001/2002 strain and other contemporary SVA strains [31]. The study revealed that SVV 001/2002 belonged to an independent evolutionary clade, while the other strains clustered within a separate clade [31]. These findings aligned with our analysis of Taiwanese SVA strains, which also demonstrated genomic evolution of SVA. To further delineate the genetic relationships among SVA strains, we constructed phylogenetic trees based on the complete genome and the four structural protein-coding regions: VP4, VP2, VP3, and VP1. The clustering patterns derived from nucleotide sequence analyses of VP1-VP3 were largely consistent with those from the full-length sequences phylogenetic tree, reinforcing the robustness of the observed phylogenetic groupings. Conversely, VP4 exhibited lower resolution with inconsistent clustering, likely due to the shorter sequence length and limited variability of the VP4 region. Therefore, VP4-based phylogenetic analysis should be interpreted with caution and regarded as supplementary to the more informative VP1–VP3-based results. These structural protein nucleotide sequence level comparisons provide additional evidence for the genetic divergence between the older Taiwanese strains (e.g., SVA-HC121223-HL-2012 in group Ia) and contemporary isolates, supporting the hypothesis of multiple introduction events or independent evolutionary events. A study analyzed the full-length sequences of historical and contemporary SVA strains and reported a 6.32% genetic divergence between them [15]. Their phylogenetic analysis revealed that most SVA strains cluster based on their geographic origins, suggesting independent evolution in different regions. However, some contemporary strains from PROC and Colombia clustered with strains isolated from the USA, indicating possible cross-regional transmission of SVA strains [15]. A comprehensive review further summarized the global distribution and evolutionary trends of SVA. They noted that historical SVA strains were primarily detected in North America, whereas contemporary SVA strains have spread globally, including the Americas and Asia [3]. The review also highlighted the genetic divergence between historical and contemporary SVA strains [3,15]. Furthermore, a study from Thailand reported that the SVA strains detected in there were closely related to Canadian strains, suggesting that international swine transport is a contributing factor in the spread of SVA [10]. The clustering of contemporary Taiwanese SVA isolates within group II highlights potential regional transmission patterns and viral evolution. Understanding these evolutionary dynamics is crucial for developing targeted control measures.

Moreover, analysis of linear B cell epitopes revealed high sequence conservation across Taiwanese and global strains, supporting their potential utility in broad-range diagnostic assays and immunogen design. Notably, most epitopes remained unchanged despite phylogenetic divergence, indicating antigenic stability. The few observed amino acid substitutions were rare and limited to individual strains, suggesting minimal impact on epitope-based recognition. These findings reinforce the suitability of conserved epitopes as reliable molecular targets. Interestingly, a substitution observed within the VP2 ^12^DRVITQT^18^ epitope was only observed in early North American isolates, including the first reported USA strain MT360258 (2002), NC011349 (2008), and one Canadian strain KY486158 (2015). This variation was absent in all other strains, including recent global and Taiwanese isolates. Such a pattern suggests that the T15I substitution may represent a variation from early SVA lineages rather than a feature of ongoing antigenic drift. Its disappearance in contemporary strains might reflect evolutionary constraints or selection pressure favoring the conserved DRVITQT motif. Similarly, a unique W71G substitution in the VP2 ^71^WTKAVK^76^ epitope was observed in only one recent Taiwanese strain (1111570), with no comparable variation detected in other strains. The high conservation of this epitope in contemporary strains further supports its potential for serological assay development.

As an emerging vesicular disease pathogen in swine, SVA has spread across major swine-producing regions in the Americas and Asia [14], with recent cases now reported in Europe. Notably, during a bilateral video conference on SVA research between VRI and the Pirbright Institute in the United Kingdom in 2024, it was highlighted that SVA cases have been detected in the UK, further emphasizing its expanding geographic distribution (UK Government News, 2024, https://www.gov.uk/government/news/seneca-valley-virus-confirmed-in-pigs-in-england, accessed on 1 November 2024) [32]. Despite its growing geographic distribution in the swine industry, information on the animal and farm-level prevalence of SVA is scant. One of the major challenges in studying the epidemiology of SVA is its tendency to result in subclinical infections, which may be unlikely to be detected without active surveillance or laboratory confirmation [15,28,33]. Therefore, establishing baseline prevalence data is crucial for enhancing our understanding of the virus’s occurrence within pig populations and its national geographic distribution.

Our study revealed distinct differences in the seroprevalence of SVA between nursery/weaned swine and finisher swine, both at the herd and animal-levels. The farm-level seroprevalence for nursery/weaned swine was 53.0% (95% CI, 47.2–58.8), while for finisher swine, it was significantly lower at 6.7% (95% CI, 4.1–10.1, *p* < 0.0001). Similarly, the animal-level seroprevalence in nursery/weaned swine was 36.2% (95% CI, 34.8–37.7), which was significantly higher (*p* < 0.0001) than the 4.6% (95% CI, 4.0–5.3) detected in finisher swine. However, herds were not significantly likely to seroconvert or become seronegative between the two sampling time points. Among the six herds classified as late seroconversion—those seronegative at the nursery/weaner stage but seropositive at the finisher stage—RRT-PCR testing was performed, and viral RNA was detected in three of these herds. This indicates that active or recent infection likely occurred between the two sampling points. The detection of SVA RNA in these herds provides additional support for the interpretation that late seroconversion reflects recent exposure post-weaning. It also underscores the dynamic nature of SVA transmission and highlights the value of integrating serological and molecular surveillance to capture the complete picture of virus circulation within herds.

The seropositive pigs in the nursery/weaner stage that tested seronegative in the finisher stage suggest possible shifts in immunity. These are likely driven by maternal antibody transfer followed by subsequent viral exposure in the environment. These findings provide valuable insight into the immune status of pigs across different stages of growth and support the hypothesis that maternal immunity plays a key role in the immunity patterns observed in Taiwanese swine, as reported in other international studies [13,34]. The observed reduction in seroprevalence with age in our study differs from the findings reported by a 2016 investigation, where the farm-level seroprevalence in pigs aged 6–26 weeks and sows aged >26 weeks in the USA were 42.7% and 75.8%, respectively [13]. That study noted in their discussion that while pigs around six weeks of age were included in their study, the presence of maternal antibodies in the youngest pigs could not be completely ruled out. This grouping approach and the potential influence of maternal immunity may have contributed to the higher overall seroprevalence reported in their study compared to our stratified analysis of different growth stages [13]. In contrast, the seroprevalence of 7.4% in finisher pigs (20 weeks or older) reported in 2018–2019 aligns closely with the low seroprevalence observed in finisher pigs in our study [28].

The stark contrast between the high sow seroprevalence reported in 2016 (75.8%) and the much lower rate observed during 2018–2019 (17.3%) may indicate a declining trend in SVA circulation in the USA swine population over time. Notably, the 2016 study was conducted shortly after the peak of SVA outbreaks in 2015, whereas the 2018–2019 study was conducted when viral transmission may have diminished. This temporal difference underscores the importance of longitudinal surveillance in tracking the epidemiological dynamics of emerging vesicular diseases. In addition to temporal factors, methodological differences may also account for some of the variation in seroprevalence estimates. The 2018–2019 study used immunofluorescence assay (IFA) for serological detection, while the 2016 study employed both ELISA and IFA with parallel interpretation of results, potentially increasing assay sensitivity. In contrast, our study used VNT, which, while more time and resource-intensive, is recommended by WOAH as the gold standard for antibody detection in other vesicular diseases of swine caused by *Picornaviridae* viruses, such as foot and mouth disease, due to its high specificity and reliability.

SVA transmission can occur through both direct contact with infected pigs and indirect contact with contaminated environments, transportation vehicles, or personnel. Breeding herds typically have higher stocking densities compared to swine aged 6–26 weeks, and this higher stocking density may facilitate more efficient viral transmission, further exacerbating the spread of SVA in these groups/environments [35]. Furthermore, sows could experience immune suppression during their reproductive cycles, which may increase their susceptibility to SVA infections. This immune suppression in sows could contribute to the higher SVA prevalence reported in these animals [36], as the compromised immune response makes them more likely to virus shedding and subsequent transmission to their piglets. This, in conjunction with persistent viral shedding, may contribute to the higher seroprevalence in sows compared to pigs aged 6–26 weeks [36]. It has been demonstrated that piglets born to SVA-infected sows acquire maternal antibodies, which can persist for up to 98 days post-birth [34]. As sows are usually kept in commercial piggeries for several years they are more likely to be exposed to infection. The persistent nature of SVA infections complicates control efforts, as sows have been shown to shed the virus for up to 60 days post-infection. In contrast, when pigs are moved to nursery/weaner pens and subsequently grower-finisher pens, they are housed in larger spaces with lower stocking density reducing their potential exposure to the virus. Additionally, the faster turnover rate of grower/fattener pigs, which are typically slaughtered around six months of age, further reduces their exposure time.

Our study also highlights significant regional variations in SVA seroprevalence. The farm-level seroprevalence in nursery/weaned swine was significantly higher in the southern region compared to the northern region (OR 3.3; 95% CI, 1.4–7.7, *p* < 0.01). Also, the animal-level seroprevalence in the central region was 1.5 times higher than that in the northern region (OR 1.5; 95% CI, 1.1–1.9, *p* = 0.0033), while the southern region exhibited even higher odds of seropositivity (OR 2.7; 95% CI, 2.2–3.5, *p* < 0.0001). For finisher swine, the highest farm-level seroprevalence was observed in the central region, where the animal-level seroprevalence was significantly higher than that of offshore islands (OR 8.5; 95% CI, 1.2–61.2, *p* = 0.0055). This regional difference may suggest that environmental or management factors, such as swine density and farm-to-farm contacts, may play a role in SVA spread. Swine farming in Taiwan is primarily concentrated in the central and southern regions, which could contribute to the higher seroprevalence observed in these areas. Previous studies have highlighted the importance of regional differences and management factors in influencing the prevalence of swine diseases [20,28]. The close contact among pigs during mixing at markets or transportation significantly increases the risk of SVA transmission. Studies from North Carolina in USA have demonstrated that the circulation of SVA is prevalent in secondary markets (slaughterhouses that purchased lower quality pigs and cull sow markets [14]). This highlighted that direct or indirect close contact between pigs, whether through mixing at markets or in densely stocked environments, facilitates the spread of the virus. Interestingly, no seropositive farms were detected from offshore islands in both the nursery/weaner stage and finisher stage. This might be a result of their geographical isolation.

Although SVA infection typically does not cause systemic disease, its vesicular lesions are clinically indistinguishable from those caused by high-consequence diseases such as FMD. The time and resources required for differential diagnosis are substantial, emphasizing the importance of studies like this in providing essential baseline data and informing the development of improved surveillance and diagnostic strategies.

## 5. Conclusions

This study confirmed the widespread presence of SVA in Taiwanese swine herds, with both farm-level and animal-level seroprevalence significantly higher in nursery/weaned swine compared to finisher swine. While this suggests that SVA is likely spreading within swine populations, the specific mechanisms of transmission were not directly investigated in this study, underscoring the need for further research to elucidate its pathways and dynamics. Although SVA infection does not typically result in clinical disease, its vesicular lesions are similar to those of notifiable diseases such as FMD. The differentiation of these conditions requires time and resources, emphasizing the importance of understanding the epidemiology of SVA. This study provides critical baseline data to support targeted surveillance and control measures, which are essential for reducing the diagnostic burden and economic costs associated with vesicular disease outbreaks in swine populations.

The observed regional differences in farm seroprevalence highlight the central and southern regions as potential hotspots for SVA transmission. Phylogenetic analyses revealed that historical Taiwanese SVA strains are closely related to early USA strains, whereas Taiwanese strains from 2018 to 2022 show a closer relationship to contemporary USA strains. Additionally, the high conservation of key B cell epitopes among Taiwanese and global SVA strains suggests that currently available or future serological assays and immunogen-based interventions may be applicable. These findings enhance our understanding of the epidemiology and molecular evolution of SVA, offering valuable insights for the development of effective strategies to mitigate its impact on the swine industry.

## Figures and Tables

**Table 1:** Primers used for full-length genome sequencing of *Senecavirus* (SVA) in this study.

Primer	Sequence (5′–3′)	Location of MN233025(nt)
SVA-1F	ATGCCCAGTCCTTCCTTTCC	18–782
SVA-1R	CGAATCGTAAACACCATTGTTCACC	18–782
SVA-2F	TACTGCCTGATAGGGCGAC	608–1408
SVA-2R	CCGTTGAGGCCTCCCT	608–1408
SVA-3F	GCCATCGACAGGTGGTACA	1290–2497
SVA-3R	TAGTCACTGGGCGAGATGTAG	1290–2497
SVA-4F	ATGGCAAGAGGGAAATTCCT	2343–3821
SVA-4R	TGGAGGAGGCGGTTCTAC	2343–3821
SVA-5F	CGCTATCTAACCAAGCTTCAG	3689–5198
SVA-5R	GTTAGGCTGTTGCATTTCCAT	3689–5198
SVA-6F	AAGTACTTCTCTGGCTCTGATACA	5049–6434
SVA-6R	AGGATGGGATTGAAACTTGG	5049–6434
SVA-7F	CTACTCTGATCATGTCTTCCAAAC	6272–7295
SVA-7R	TTTTTTTTTTTTTTTTCCCTTTTCTGTCCC	6272–7295

**Table 2:** Farm-level seroprevalence of SVA in swine at the nursery/weaner stage across different regions of Taiwan.

Regions	Number of Test-Positive Farms/Total Number Tested	Farm-Level Seroprevalence (95% CI)	OR (95% CI)	*p*-Value	Number of Test-Positive Animals/Total Number Tested	Animal-Level Seroprevalence (95% CI)	OR (95% CI)	*p*-Value
Northern	10/27	37.0% (19.4–57.6)	1.0	-	97/405	24.0% (19.9–28.4)	1.0	-
Central	58/123	47.2% (38.1–56.4)	1.5 (0.6–3.6)	0.3969	573/1824	31.4% (29.3–33.6)	1.5 (1.1–1.9)	0.0033 *
Southern	88/134	65.7% (57.0–73.7)	3.3 (1.4–7.7)	0.0087 *	931/2009	46.3% (44.1–48.6)	2.7 (2.2–3.5)	<0.0001 *
Eastern	3/8	37.5% (8.5–75.5)	1.0 (0.2–5.2)	1	33/120	27.5% (19.7–36.4)	1.2 (0.8–1.9)	0.4702
Offshore islands	0/8	0.0 (0.0–36.9)	-	0.0734	0/150	0.0% (0.0–2.4)	-	<0.0001 *
Total	159/300	53.0% (47.2–58.8)	-	-	1634/4508	36.2% (34.8–37.7)	-	-

* Indicates a statistically significant difference (*p* < 0.05) compared to the reference regions, which is the northern region for farm-level and animal-level seroprevalence.

**Table 3:** Farm-level seroprevalence of SVA in swine at the finisher stage across different regions of Taiwan.

Regions	Number of Test-Positive Farms/Total Number Tested	Farm-Level Seroprevalence (95% CI)	OR (95% CI)	*p*-Value	Number of Test-Positive Animals/Total Number Tested	Animal-Level Seroprevalence (95% CI)	OR (95% CI)	*p*-Value
Northern	2/27	7.4% (0.9–24.3)	1.45 (0.3–7.4)	0.6473	10/380	2.6% (1.3–4.8)	3.5 (0.5–27.7)	0.304
Central	11/123	8.9% (4.6–15.4)	1.78 (0.7–4.8)	0.3286	102/1667	6.1% (5.0–7.4)	8.5 (1.2–61.2)	0.0055 *
Southern	7/134	5.2% (2.1–10.5)	1.0	-	78/1852	4.2% (3.3–5.2)	5.7 (0.8–41.4)	0.0596
Eastern	0/8	0 (0.0–36.9)	-	1	1/115	0.9% (0.0–4.7)	1.1 (0.1–18.4)	1
Offshore islands	0/8	0 (0.0–36.9)	-	1	1/131	0.8% (0.0–4.2)	1.0	-
Total	20/300	6.7% (4.1–10.1%)	-	-	192/4145	4.6% (4.0–5.3%)	-	-

* Indicates a statistically significant difference (*p* < 0.05) compared to the reference regions, which is the southern region for farm-level seroprevalence and the offshore islands for animal-level seroprevalence.

## Data Availability

The data supporting the findings of this study are available from the corresponding authors upon request.
